# Wild redfronted lemurs (*Eulemur rufifrons*) use social information to learn new foraging techniques

**DOI:** 10.1007/s10071-012-0477-y

**Published:** 2012-03-18

**Authors:** Anna Viktoria Schnoell, Claudia Fichtel

**Affiliations:** 1 Behavioral Ecology and Sociobiology Unit, German Primate Center, Kellnerweg 4, 37077 Göttingen, Germany; 2 Courant Research Center “Evolution of Social Behavior”, University of Göttingen, Göttingen, Germany

**Keywords:** Eulemur rufifrons, Traditions, Conformity, Directed social learning, Culture, Scrounging

## Abstract

Recent research has claimed that traditions are not a unique feature of human culture, but that they can be found in animal societies as well. However, the origins of traditions in animals studied in the wild are still poorly understood. To contribute comparative data to begin filling this gap, we conducted a social diffusion experiment with four groups of wild redfronted lemurs (*Eulemur rufifrons*). We used a ‘two-option’ feeding box, where these Malagasy primates could either pull or push a door to get access to a fruit reward to study whether and how these two behavioural traits spread through the groups. During a pre-training phase, two groups were presented with boxes in which one technique was blocked, whereas two groups were presented with unblocked boxes. During a subsequent unconstrained phase, all four groups were confronted with unblocked boxes. Nearly half of the study animals were able to learn the new feeding skill and individuals who observed others needed fewer unsuccessful task manipulations until their first successful action. Animals in the two groups with pre-training also discovered the corresponding alternative technique but preferred the seeded technique. Interestingly, animals in the two groups without pre-training discovered both techniques, and one group developed a group preference for one technique whereas the other did not. In all groups, some animals also scrounged food rewards. In conclusion, redfronted lemurs appear to use social information in acquiring a novel task, and animals in at least in one group without training developed a group preference for one technique, indicating that they have the potential to develop behavioural traditions and conformity.

## Introduction

Recent research in animal behaviour has focused on the mechanisms underlying the spread of traditions in animal societies (Laland and Janik 2006; Whiten and van Schaik^ 2007^; Laland and Galef^ 2009^). Traditions are considered to be distinctive behaviours that differ within or between populations, are shared among members of a group and characterized by their persistence over time, and, most importantly, by being acquired through social learning (Fragaszy and Perry 2003). The strongest evidence for traditions in vertebrates has so far been found in birdsong dialects (Catchpole and Slater 1995), but also in various other behavioural contexts, including food processing techniques (primates: Kawai 1965; Whiten et al. 1999; van Schaik et al. 2003; Perry 2009; cetaceans: Rendell and Whitehead 2001; Krützen et al. 2005; birds: Hunt and Gray 2003), affiliative behaviours (primates: Whiten et al. 1999; Perry et al. 2003; Santorelli et al. 2011), communication (cetaceans: Janik and Slater 1997; primates: Fichtel and Kappeler 2011) and mating site preferences (fish: Warner 1988).

Many insights into animal culture have derived from inter-population comparisons of behavioural traits in wild populations (Sugiyama 1997; Whiten et al. 1999; Rendell and Whitehead 2001; van Schaik et al. 2003; Boinski et al. 2003; Perry 2009; Krützen et al. 2005). Even though these studies have revealed rich repertoires of behavioural variation within and between populations, the origin of these behaviour patterns remains unclear because it is difficult to assess by field observations alone whether a trait was acquired through social or individual learning. Social learning is by definition the essential mechanism for the formation of traditions, as it is necessary for diffusion and maintenance of intra-group specific behaviours. It is defined as ‘learning that is influenced by observation of, or interaction with, another animal (typically a conspecific) or its products’ (Heyes 1994).

Several recent studies have demonstrated social learning and the spread of new behaviours in animals by introducing different feeding techniques (pigeons (*Columba livia*): Lefebvre 1986; white-throated magpie jays (*Calocitta formosa*): Langan 1996; chimpanzees (*Pan troglodytes*): Whiten et al. 2005; meerkats (*Suricata suricatta*): Thornton and Malapert 2009; banded mongoose (*Mungos mungo*): Müller and Cant 2010; and orangutans (*Pongo pygmaeus, P. abelii*): Dindo et al. 2011). For example, by introducing an artificial feeding box that could be opened by using two different techniques to groups of captive chimpanzees, it could be demonstrated that the respective foraging technique was learned socially by other group members, leading to social diffusion within groups, which, in turn, was subject to conformity (Whiten et al. 2005). In a broader sense, social conformity is considered as an adoption of the group’s norm, despite being in principle able to behave differently (Whiten et al. 2005), or over-riding of individually learned by socially acquired information (Galef and Whiskin 2008; Dindo et al. 2009, but see Laland 2004). Thus, social conformity represents a strong indirect indicator for social learning. Subsequent research has revealed that conformity is not unique to chimpanzees and humans (Whiten and van Schaik  2007), but is also present in other mammals, such as brown capuchins (*Cebus apella*; Dindo et al. 2009), guppies (*Poecilia reticulata*; Day et al. 2001) or Norway rats (*Rattus norvegicus*), which even learned to suppress their personal knowledge about toxic or safe food as well as good or bad tasting food items by observing others (Galef and Whiskin 2008).

The advantage of social diffusion experiments is that animals are tested at a group level, that is, in a situation similar to the one in which social learning would normally occur in the wild (Whiten et al. 2005; Whiten and Mesoudi 2008). Whereas such experiments in captivity have the potential to reveal whether behavioural traits in groups are subject to individual modification or social transmission, field studies can provide an ecologically more valid picture (reviewed in Reader and Biro 2010). Because animals in the wild have to manage their time and energy budgets carefully (Parker 1990), social diffusion might be of vital importance. Social learning can help to save energy because individuals do not have to learn certain behaviours by themselves but can instead observe and copy/imitate others (Laland 2004). However, there is also always a risk that individuals might gather incorrect or out-dated information, which would increase the costs of learning considerably (Parker 1990; Kendal et al. 2005; Laland et al. 2005). In addition, identification of social learning in free-living animals is a crucial step in elucidating the interaction between biological and cultural evolution (Kendal et al. 2010a). Thus, field studies of social learning can provide important insights into the nature of traditions.

However, only a few studies have examined the spread of new foraging skills under natural settings in birds and mammals experimentally, including pigeons (Lefebvre 1986), magpie jays (Langan 1996), keas (*Nestor notabilis*; Gajdon et al. 2004), meerkats (Thornton and Malapert 2009), wild banded mongooses (Müller and Cant 2010) and primates (Pesendorfer et al. 2009; Kendal et al. 2010b; van de Waal et al. 2010; reviewed in Reader and Biro 2010). Interestingly, a comparison between captive and wild pigeons revealed that the level of social diffusion of a new foraging task was higher in wild compared to captive pigeons, probably due to stronger selective pressure on the development of efficient foraging skills (Lefebvre 1986), emphasizing the importance of social learning studies in wild populations (Kendal et al. 2010a).

Field studies on social learning in wild primates have revealed that ringtailed lemurs (*Lemur catta*) learn socially but only within subgroups (Kendal et al. 2010b). Vervet monkeys (*Chlorocebus aethiops*) exhibited higher homogeneity in the task if a skilled demonstrator was present and therefore seem to use information provided by others (van de Waal et al. 2010). However, common marmosets (*Callithrix jacchus*), in which the maintenance/conformity of a learned skill was studied as an indirect indicator for social learning, did not adjust their behaviour to that of the groups majority and presumably did not rely on the use of social information (Pesendorfer et al. 2009).

In order to add to these few studies on social learning in wild primates, we studied social diffusion of a two-choice foraging technique in wild redfronted lemurs (*Eulemur rufifrons*). Even though the brain size of Malagasy lemurs is relatively smaller compared to that of Old and New World monkeys (Armstrong 1985), and despite some early doubts about their intelligence (Jolly 1966), the ability to form behavioural traditions has been shown in the wild (Fichtel and Kappeler 2011). So far, however, the ability to learn socially has been demonstrated in lemurs in captive and semi-free ranging settings (Kappeler 1987; Anderson et al. 1992; Hosey et al. 1997; Stoinski et al. 2011; reviewed in Fichtel and Kappeler 2010), but only one study has been conducted on wild lemurs to date (Kendal et al. 2010b).

Since ringtailed lemurs show restricted social tolerance towards close kin (Jolly and Pride 1999) and social tolerance has an impact on social learning opportunities (Coussi-Korbel and Fragaszy 1995; van Schaik et al. 1999; Reader and Biro 2010), the restricted spread of innovations among subgroups observed by Kendal et al. (2010b) might be due to their hierarchically organized social structure. Thus, comparative studies in wild lemurs with different social structure may illuminate a potential influence of social tolerance on social learning in natural settings.

Redfronted lemurs are organized into groups with a relatively egalitarian social structure; they do not exhibit a linear dominance hierarchy nor is one sex consistently dominant over the other (Pereira et al. 1990; Ostner and Kappeler 2004). In addition, they exhibit high levels of social affinity (Pereira and Kappeler 1997), which allows individuals to spend time in close proximity to others, facilitating directed social learning (Coussi-Korbel and Fragaszy 1995). The aim of this study was to investigate the spread of two different foraging techniques of an artificial fruit task under natural conditions. We asked (1) whether wild redfronted lemurs can learn a new foraging technique, (2) if so, whether they learn it individually or socially and (3) whether they adapt their behaviour to the majority of the group, thereby exhibiting conformity. To detect social learning, we also used a method for identifying social learning in natural conditions, the network-based diffusion analysis (Franz and Nunn 2009), for which we additionally conducted animal focal observations to establish a social network.

## Methods

### Study site and subjects

This study was conducted at the research station of the German Primate Center in Kirindy Forest, Western Madagascar (Kappeler and Fichtel 2012a). Data collection took place between September and December 2009, which corresponds to the transition between dry and rainy seasons. We studied 37 individuals living in four habituated groups (Table 1). As part of a long-term study, all subjects have been individually marked with nylon collars and are well habituated to human presence (Kappeler and Fichtel 2012b). Kin relationships were known, except for some immigrant males. Redfronted lemurs were naïve with respect to the experimental protocol and had no experience with any food not growing naturally in the forest.

**Table 1:** Composition of the study groups and corresponding conditions

Condition (group)	Pull group (A)	Push group (J)	Open group (B)	Open group (F)	In total
Number of adult males	6	4	5	4	19
Number of adult females	3	2	2	3	10
Number of juvenile males	2	0	1	2	5
Number of juvenile females	1	2	0	0	3
Total number of subjects	12	8	8	9	37

The pull and push condition received a training whereas the open condition did not receive any training

### Experimental apparatus

**Fig. 1:**
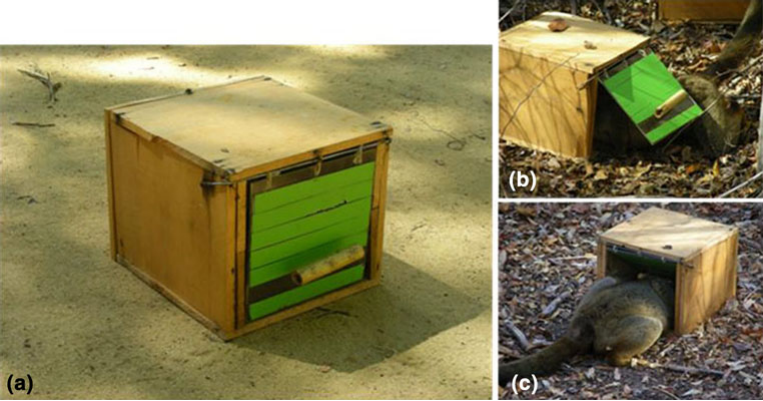
Experimental apparatus: The feeding box (**a**) offered two distinctive techniques for extracting reward—the door could either be pulled (**b**) or pushed (**c**)

We used a feeding box similar to the one used by Bugnyar and Huber (1997) in a laboratory study with common marmosets and Pesendorfer et al. (2009) on the same species in the field. The box was constructed of wood and measured 16 × 20 × 20 cm (Fig. 1). The front side was open, but covered by a 15 × 15 cm flap door made of plexiglas that was covered with tape and equipped with a handle to move the door. The feeding box could be opened by two different techniques: by pulling or pushing the door (Fig. 1). Since both movements were directed to the same location, that is, the door, simple social learning mechanisms like local or stimulus enhancement should not account for copying of pulling versus pushing (McGrew 1998; Huber et al. 2009). Both actions were likely to have the same degree of difficulty as the data on wild common marmosets showed similar rates of pulling versus pushing actions for control groups (Pesendorfer et al. 2009).

### Experimental set-up and procedure

**Fig. 2:**
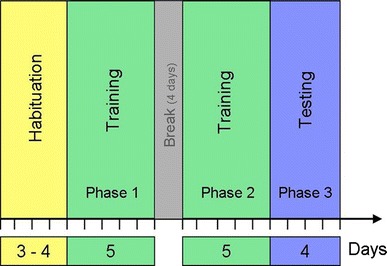
Experimental procedure: Each group passed through habituation, training and testing. Data were collected in phase 1, phase 2 and phase 3

Animals were first habituated to novel fruits (oranges and mangos) used as a reward and the feeding box for 3–4 days (Fig. 2). Afterwards, they were assigned to three different conditions for the training phases—two groups were offered only one of the two techniques—to open the door either by pulling (condition: pull group) or by pushing (condition: push group) (Fig. 2). Two additional groups could freely choose between both techniques from the beginning (condition: open groups). The first two training phases lasted between 7 and 10 days with a break of 4 days in between. The pull group and the two open groups were trained for 10 days, whereas the push group was trained for only 7 days because half of the group members were already able to perform the task at this point. After the training, all groups were confronted with unconstrained boxes to test whether redfronted lemurs learn to open the box by the alternative technique, and if so, whether they nevertheless continue opening the box mainly with the originally learned technique.

To avoid monopolization of the box by socially powerful individuals, we presented two to three boxes simultaneously during the training and unconstrained conditions. The boxes were filled with several pieces of oranges or mangos. Feeding boxes were presented when a group was resting or feeding, preferentially when the animals gathered on or near the ground. They were placed on open spots on the ground so that all interactions at the boxes could be video-taped. Each group was tested once per day either in the morning between 07:00 and 11:30 a.m. or in the afternoon between 14:00 and 17:00 p.m. in a counterbalanced order. The experiment started when an individual approached a box within a 1-m radius and ended when the last animal left the 10-m radius. Sessions lasted between 14 and 58 min (mean ± SD: pull group = 35.6 ± 12.0 min; push group = 24.8 ± 8.7 min; open group B = 22.9 ± 8.7 min; open group *F* = 16.9 ± 4.5 min).

In addition to the video recordings, we noted every second minute the position and distance of all individuals gathering within a range of 10 m around the boxes. We also recorded whether individuals observed others, that is, whether their head turned in the direction of the boxes while another individual was manipulating the box. This sampling method was chosen because it was the shortest feasible time interval to protocol position, distance and looking directions of all group members present within a radius of 10 m around the boxes. The 10-m radius was chosen because the average group spread of redfronted lemurs is 15 m (Pyritz et al. 2010). We calculated the percentage of time spent observing others for each individual by dividing the number of scans it spent observing others by the total number of scans the individual spent in the 10-m radius.

### Data analyses

Video sequences of the experiments were recorded with a Sony video-camera (DCR-PC105E PAL) installed on a tripod. Recordings were analysed with Adobe CS 3 Premiere Pro. The identity and sex of individuals at the test location as well as a set of other variables describing interactions with the boxes and conspecifics were recorded. We noted the numbers of aggressive events at the boxes and calculated a relative aggression score as an index of monopolization of the feeding box by dividing the number of aggressive interactions initiated by each individual by the total number of aggressive interactions initiated and received within a 1-m radius of a box. To investigate whether females and males differ in aggression scores, we used the proportion of initiated aggressive and total number of aggressive interactions per individual as the response term in a Generalized Linear Mixed Model (GLMM) with binomial errors (Crawley 2007). Sex was used as a fixed factor and individual identity as a random factor. A maximum likelihood ratio tests was used to test the final model with fixed factors against the null model including only the intercept and random factors (Faraway 2006).

We measured the duration individuals manipulated a box with their hands or nose. To calculate the efficiency in retrieving food rewards, the number of successful actions was divided by the number of total actions performed at the door for a given individual. A successful action was defined as moving the door and retrieving a reward, whereas an unsuccessful action was defined as manipulating the door but not retrieving a reward. Because some individuals (*N* = 4) performed fewer than 6 actions at the feeding box in phase 3, we considered them as exhibiting a preference when the majority of actions were performed with the same technique, that is, number of actions the individual performed using the preferred technique divided by the total number of actions (see Dindo et al. 2009). In cases in which individuals performed more than 6 actions at the feeding box, we used a binomial test to test for a preference for one technique. Preference scores as well as outcomes of the binomial tests are presented with individual acronyms, indicating the individual’s social group, sex and the first three syllables of its name, for example AFCor stands for the female Corsica in group A. Individuals that performed fewer than 3 actions in phase 3 were excluded from the analysis of preference. To calculate a scrounging preference, all scrounging actions were divided by the total number of actions at the box. Correlation analyses as well as non-parametric two-tailed tests were conducted in SPSS 17.0. In order to examine whether the efficiency in retrieving food rewards changed over the experimental phases, a permutation test for repeated measurements with missing values was used (Mundry 1999), because not all individuals manipulated the boxes in each phase.

To test the influence of the pre-training on learning success, we calculated a Generalized Mixed Linear Model. We used the number of unsuccessful task manipulations until the first success as dependent variable, pre-training (yes or no) as a fixed factor and group identity as a random factor. To assess the influence of kinship or social bonds on observing other individuals manipulating the box, the rate of observations (number of scans individuals spent observing others/total observation time) a given individual spent watching each other individual manipulating the box was calculated. The rate of observing others manipulating the box was arc sin-square root transformed to calculate a Linear Mixed Model (LMM). We used the rate of observing others manipulating the box as the dependent variable. Kinship and sex were used as fixed factors, and individual identity was used as a random factor. Because kinship and social bonds (mean duration of affiliative interactions) were positively correlated (Spearman rho: ρ = 0.25, *p* < 0.001, *n* = 128), we included only kin as a fixed factor.

To investigate whether one of the open groups showed a preference of one over the other technique, we used the proportion of push and pull actions performed in each session by a given individual as the response term in a Generalized Linear Mixed Model (GLMM) with binomial errors (Crawley 2007). Group was used as a fixed factor, and individual identity as a random factor. For both models (LMM and GLMM), we used maximum likelihood ratio tests to test the final model with fixed factors against the null model including only the intercept and random factors (Faraway 2006). Models were calculated in R 2.8.1 (Development Core Team 2009).

### Behavioural observations

To establish a social network, all 37 individuals were each observed for 2.5 h (in total 92.5 h) by conducting focal animal observations (Altmann 1974). Animals were observed on different days for 30 min focal observation periods equally spread between 06:00 and 18:00 h. We recorded all affiliative, affinitive and aggressive interactions. Resting in contact, resting within a 1-m radius with another individual and grooming were considered as affiliative interactions (modified after Pereira and Kappeler 1997). Symmetrical social networks were constructed on the mean durations of affiliative interactions during 30 min observations (grooming, resting in contact and resting together). Kinship was assigned by classifying all animals having a kinship relation of ≥0.25 as kin and all others as non-kin.

### Network-based diffusion analysis

We also conducted a network-based diffusion analysis (NBDA; Franz and Nunn 2009) to test the influence of social learning on the task. The NBDA tests for social learning by including the social aspect of group structure. It takes into account the social learning opportunities between pairs of individuals that a social network offers, as described by the theory of directed social learning (Coussi-Korbel and Fragaszy 1995). Social learning is inferred if the order and time at which individuals first solve the task matches the social network. In this study, we used affiliative behaviour as a proxy for learning opportunity, assuming that individuals learn preferentially from conspecifics with whom they spent more time in close proximity and interact affiliatively. For our data, we used the extended version of the NBDA, which takes into account the fact that under natural conditions, it is unlikely that animals will learn by social means alone. It therefore compares the fit of a model of social and asocial learning as well as a model of pure asocial learning to the actual diffusion. Model selection was based on the Akaike information criterion (AIC). Calculations were conducted with R 2.8.1.

## Results

### Learning behaviour

Of 37 subjects of the four study groups, 36 explored the feeding boxes. In the two groups with pre-training, 12 out of 20 individuals manipulated the box and 10 of them successfully. In the pull group, 6 out of 12 individuals manipulated the box and 4 individuals acquired the task during the training phases 1 and 2. Overall, they conducted on average (mean ± SD) 66.3 ± 77.3 successful actions (Table 2). In the push group, 6 out of 8 individuals conducted actions at the box, 5 animals acquired the task during the training phases 1 and 2, and 1 animal acquired the task in phase 3. Overall, they performed on average (mean ± SD) 39.5 ± 23.4 successful actions (Table 2).

**Table 2:** Average number of successful actions animals performed in the different experimental phases, average number of unsuccessful task manipulations for each group and average scrounging preference per group

	Pull group (*N* = 4)	Push group (*N* = 6)	Open group B (*N* = 4)	Open group F (*N* = 4)
Successful actions performed at the box (mean ± SD)				
Phase 1 + 2	42.5 ± 48.8	29.4 ± 27.8	41.7 ± 29	82.3 ± 36.7
Phase 3	23.8 ± 28.9	15 ± 11.3	17.3 ± 14.4	30 ± 25.6
Unsuccessful task manipulations (median (IQR))				
	4.5 (2.5)	3 (4)	13 (5.5)	10 (7.5)
Scrounging preference (median (IQR))				
	44.5 % (72.7 %)	17.4 % (7.7 %)	21.3 % (25.4 %)	22.7 % (23.1 %)

In the two open groups in which the feeding apparatus was not constrained from the beginning, 10 out of 17 individuals manipulated the boxes (group B: 6 out of 8 individuals; group F: 4 out of 9), 8 of them successfully. In open group B, 4 out of 6 individuals learned the task (3 during the training phases 1 and 2, and 1 in phase 3). Overall, they performed on average (mean ± SD) 44.3 ± 46.0 successful actions (Table 2). In open group F, 4 individuals performed the task successfully and conducted on average (mean ± SD) 91.8 ± 71.1 successful actions (Table 2).

The inventors, that is, the first individual in each group learning the task by trial and error (Kendal et al. 2009), were young individuals (1–2 years) in three of the study groups: In the pull group and in the open group F, inventors were juvenile males (AMKor: 1 year; FMCas: 1 year) and in the push group a young female (JFMal: 2 years). In the open group B, an adult female was the first individual to succeed (BFSip: 12 years). On average, subjects needed 7.1 ± 6.2 (*n* = 18) unsuccessful task manipulations until their first successful operation (Table 2). Learning success, that is, the number of unsuccessful task manipulations until first success, differed across the conditions with individuals of the open groups needing more manipulations than the other two groups (GLMM, χ^2^ = 11.55,* df* = 1, *p* < 0.001, Table 2, 3). Interestingly, there was a sex difference in learning success with only 33 % of males, but 77 % of females learning to extract rewards from the box (Mann–Whitney* U* test:* Z* = −2.498, *p* = 0.03, *n* = 37). Efficiency of performing the task did not change over the three experimental phases (repeated measurement test: *p* = 0.5, *n* = 18).

**Table 3:** Average aggression score for males and females and average preference for males and females observing individuals of the same or other sex

	Males (*N* = 24)	Females (*N* = 13)
*Aggression score (median (IQR))*		
	0.4 (0.6)	0.4 (0.7)
*Preference for observing individuals of the same or other sex (median (IQR))*		
Males	45 % (27 %)	56 % (27 %)
Females	50 % (26 %)	51% (26 %)

Individuals aggressively defended the boxes, and as a result animals with higher aggressive scores spent more time in contact with the boxes (Spearman rho: ρ = 0.55, *p* < 0.001, *n* = 37). Females and males did not differ in aggression scores (Tables 3, 5; GLMM, χ^2^ = 4.66,* df* = 1, *p* = 0.49). There was no difference in number of aggressive events across the conditions (Kruskal–Wallis test:* df* = 2, *p* = 0.95; median_pullgroup_ = 5 events, IQR = 10.0; median_pushgroup_ = 5 events, IQR = 16.25; median_opengroup_ = 6 events, ICR = 0.0) and none between the four groups (Krukal–Wallis test:* df* = 3, *p* = 0.74; median_opengroup F_ = 6 events, IQR = 9.0; median_opengroup B_ = 15 events, IQR = 15.0). Although some individuals defended the box, others scrounged, that is, getting access to the reward while others opened the door. In the pull group, five individuals scrounged at least once, in the push group six animals, in the open group B six animals and in open group F four animals (average scrounging preference per group see Table 2).

### Social learning

**Fig. 3:**
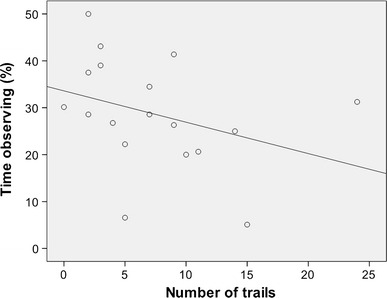
Spearman rho correlation between percentage of observing others performing the task and number of trails until first success

The percentage of time individuals spent observing other group members manipulating the feeding box until their own first successful action was negatively correlated with learning efficiency (Fig. 3; Spearman rho: ρ = −0.50, *p* = 0.03, *n* = 18). Thus, redfronted lemurs observing others performing a task used fewer unsuccessful actions while learning a task. There was no preference for observing individuals of the same or other sex (Table 3; Wilcoxon test: males: *Z *= −1.36, *p* = 0.17; females: *Z* = −0.22, *p* = 8.24). Interestingly, redfronted lemurs observed related individuals less often than non-related individuals (Table 5; LMM, χ^2^ = 11.57,* df* = 2, *p* < 0.01; median_noKin_ = 42 %, IQR = 46.8; median_kin_ = 21 %, IQR = 80.5), but sex of the observer had no effect (*t* = −0.797, *p* = 0.43, *n* = 128).

To apply the network-based diffusion analysis (NBDA), we calculated social networks for each study group, using average durations of affiliative interactions. The extended version of the analysis (asocial versus asocial and social learning model) revealed a better fit for the asocial model than the asocial and social model in both the pull group and the push group (Table 4). For the two open groups, however, the analysis did not reveal a significantly better fit of one model over the other one (Table 4).

**Table 4:** Results of the extended network-biased analysis (eNBDA)

eNBDA	Asocial model		Social and asocial model	
	AIC	Akaike probability (%)	AIC	Akaike probability (%)
Pull group^a^	29.904	73.11	31.904	26.90
Push group^a^	33.157	73.11	35.157	26.90
Open group F	25.822	70.63	27.577	29.37
Open group B	9.348	45.66	9.00	54.34

The AICs are calculated by fitting the data to an asocial model (left column) or to a social and asocial model (right column)

^a^Indicates a better fit of one model over the other one

### Group preferences

**Fig. 4:**
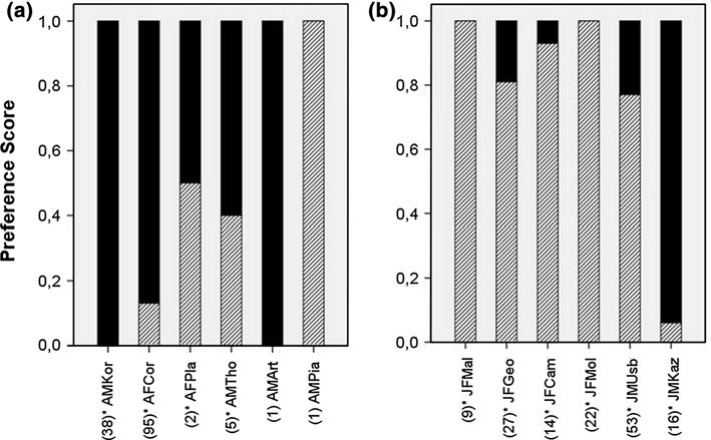
Preference scores of pulling (*black bars*) and pushing (*striped bars*) during the third phase with unconstrained boxes of **a** the pull group and **b** the push group

In the third phase of the experiment, all groups were confronted with unconstrained boxes. In the pull group, only 3 out of 6 animals carried out 3 or more actions and were therefore used for further analysis (Fig. 4). All of them used the pulling technique more often than the pushing technique (Fig. 4; binomial test: AFCor: *p* < 0.01; AMKor: *p* < 0.01; AMTho: preference score = 60 %). In the push group, 5 animals kept the originally learned technique (Fig. 4; binomial test: JFCam: *p* < 0.01; JFGeo: *p* < 0.01; JFMal: *p* < 0.01; JFMol: *p* < 0.01; JMUsb: *p* < 0.01) and 1 individual showed a preference for the alternative technique (binomial: JMKaz: *p* < 0.01). Individuals of both groups preferred the seeded technique over the alternative one (Fig. 4; binomial test: *n* = 9, *p* = 0.04; median preference_seeded_ = 87 %, IQR = 23.0; median preference_non-seeded_ = 13 %; IQR = 23.0), although 6 of them also discovered the other technique.

**Fig. 5:**
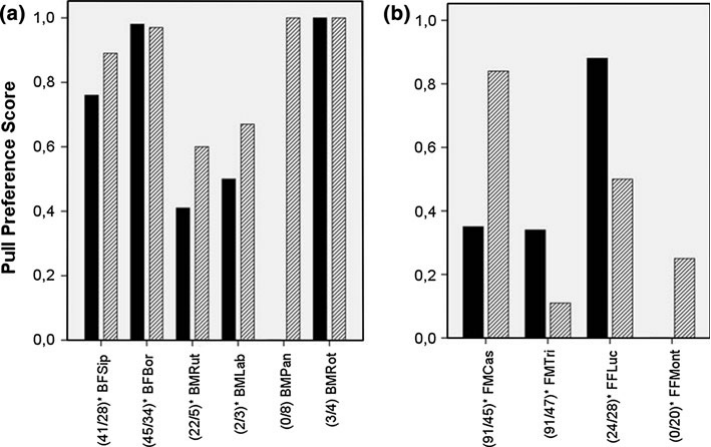
Pull preference scores in the first and third phase of **a** the open group B and **b** the open group F (1. number = n of total actions in phase 1, 2. number = n of total actions in phase 3)

In the open group F, 2 individuals showed a preference for pushing (Fig. 4; binomial test: FFMont: *p* = 0.04; FMTri: *p* < 0.01), 1 for pulling (Fig. 4; binomial test: FMCas: *p* < 0.01) and another one showing no preference (Fig. 4; binomial test: FFLuc: *p* = 1.0). Thus, there was no clear preference for one technique over the other in this group, although the inventor (FMCas) showed a push preference of 65 % in phase 1. In the open group B, all 6 subjects performed more pull than push actions (Fig. 5; binomial: BFBor: *p* < 0.01; BMPan: *p* = 0.01; BFSip: *p* < 0.01; BMLab: preference score = 67 %; BMRot: preference score = 100 %; BMRut: preference score = 60 %) and therefore preferred the pulling over the pushing technique (Fig. 5; binomial test: *n* = 6, *p* = 0.03). The inventor in open group B (BFSip) showed a pull preference of 76 % in phase 1. Individuals of the open group B exhibited a clear preference for the pulling over the pushing technique in comparison with group F (Table 5; GLMM, χ^2^ = 5.85,* df* = 1, *p* < 0.05).

**Table 5:** Parameter estimates for the linear mixed model (LMM) on determinants of observation rates and the generalized mixed linear model (GLMM) on the difference in the proportion of push and pull actions in the open groups during the unconstrained phase

Model	Response variable	Random factors	Fixed factors	Estimate	SE	*p* value
*GLMM*	Proportion of initiated and total number of aggressive interactions	Individual	Intercept	−0.72	0.18	<0.001
			Sex	−0.2	0.29	0.49
*GLMM*	Number of unsuccessful task manipulations until first success	Group identity	Intercept	2.37	0.11	<0.001
			Pre-training	−0.94	0.19	<0.001
*LMM*	Rate of observing others	Individual	Intercept	0.42	0.02	<0.001
			Sex	−0.03	0.04	0.43
			Kin	0.13	0.04	<0.001
*GLMM*	Proportion of pull and push actions	Individual	Intercept	−2.03	0.59	<0.001
			Group	2.36	0.86	<0.01

## Discussion

Our study revealed that wild redfronted lemurs are able to learn new foraging techniques and that the use of social information facilitated the acquisition of the task. More than half of the individuals who learned to open the box with one technique also discovered the alternative technique. The two groups with the seeded technique mainly preferred the originally trained technique, whereas one of the two open groups exhibited a clear preference for the pulling technique. Thus, this group appears to prefer the technique that was used by the majority of the group. As in other species, some individuals also scrounged to get access to food rewards (Fragaszy and Visalberghi 1990; Bugnyar and Huber 1997; Caldwell and Whiten 2003).

### Social learning

All individuals except one male explored the feeding boxes. Redfronted lemurs were highly motivated to find and exploit new food sources, which might be due to the fact that we conducted the experiments in the dry season when food and water are rare (Scholz and Kappeler 2004). Nearly half of the subjects who actually manipulated the box successfully learned to perform the task in this field setting. Although they are organized in a fairly egalitarian social system, several individuals were able to prevent others from interacting with the boxes, and therefore some individuals did not have a chance to perform actions at the boxes. In captive brown lemurs (Anderson et al. 1992) and rhesus macaques (*Macaca mulatta*; Drea and Wallen 1999), some individuals also prevented others from interacting with a feeding box, but passive individuals were able to perform the task successfully when the dominant animals were removed. Because we could not remove individual redfronted lemurs, we cannot know whether these individuals did not learn the task, or whether they did not manipulate the box to avoid aggression.

Individuals who were tested with the unconstrained boxes needed more unsuccessful manipulations until they could successfully open the box, compared to individuals who were trained with one of the two techniques. Thus, offering two possibilities for opening the foraging box required more time for learning and might be more difficult. Interestingly, more females than males learned the task. Similarly, in ringtailed lemurs only adult females and none of the adult males acquired a new behavioural trait, probably due to female dominance in this species (Kappeler 1987). However, redfronted lemurs lack female dominance (Pereira and Kappeler 1997), and we did not find a sex difference in aggressiveness. Because some of the females had just given birth and were lactating, they may have had a higher motivation to learn the task than males due to their increased nutritional needs (Randolph et al. 1977; Tarnaud 2006).

Although the exact learning mechanism could unfortunately not be determined with this experimental setting, redfronted lemurs who observed others manipulating the box for a longer time required fewer manipulations at the box until their first success. Thus, they appeared to use information available from conspecifics interacting with the box for dealing more efficiently with the task (Boogert et al. 2008). The network-based diffusion analysis suggests, however, that redfronted lemurs learned the task individually. Similar results have been found in ringtailed lemurs, in which the NBDA also did not pick up the transmission along the social network (Kendal et al. 2010b). However, in ringtailed lemurs, social learning was only detected on a subgroup level of 2–3 individuals, which might explain the low power of the NBDA. Similarly, small sample sizes for constructing the social network might explain why the NBDA could not detect social learning in our study. However, as redfronted lemurs exhibit a rather egalitarian social structure, the NBDA might not have detected social learning due to a lack of strong differences in social connections.

In contrast to our findings, new foraging skills spread in ringtailed lemurs only among small sub-groups (Kendal et al. 2010b). Because the two species differ in their social structure, with redfronted lemurs exhibiting a relatively egalitarian structure and ringtailed lemurs exhibiting clear dominance hierarchies (Jolly 1966; Kappeler 1990) with restricted social tolerance towards close kin (Jolly and Pride 1999), differences in the spread of new innovations can be explained by the degree of social tolerance between the species. The social structure of a society appears to have a major impact on social learning opportunities (Coussi-Korbel and Fragaszy 1995; Reader and Biro 2010), because it influences the level of social tolerance among group members, the diversity of contacts (Thierry et al. 2000; Butovskaya 2004) and proximity between animals, allowing close observation and hence social learning of other’s activities (van Schaik et al. 1999). Thus, the differences in the spread of new foraging techniques between wild redfronted and ringtailed lemurs emphasize the fact that comparative studies of species exhibiting different social structures can provide important insights into the social dynamics facilitating or inhibiting social learning mechanisms.

### Conformity in redfronted lemurs?

Interestingly, individuals of one of the open groups acquired both techniques but developed a preference for one technique, whereas the other unconstrained group did not. Thus, redfronted lemurs did not generally prefer one technique over the other, suggesting that social learning was involved in building up a preference. This finding stands in contrast to the results of similar experiments with common marmosets (Pesendorfer et al. 2009), where no general preference for one of two techniques was found in groups without training. Because marmosets are quite manipulative (Voelkl and Huber 1999; Yamamoto et al. 2004; Dell’Mour et al. 2009), they may have achieved the technique easily by individual learning and did not necessary rely on social learning. Lemurs, in contrast, have limited dexterity (Torigoe 1985) due to the lack of a precision grip (Holtkötter 1997). Therefore, opening a feeding box might present a bigger challenge for them, which could require higher levels of social learning and may explain the differences between the redfronted lemurs and common marmosets.

In general, relatedness facilitates, whereas aggression hampers social learning (Fragaszy and Visalberghi 1990; Schwab et al. 2008). In our study, however, redfronted lemurs observed unrelated individuals more often than related individuals, and the two open groups did not differ in the amount of aggression performed at the boxes. Theoretical studies suggest that individuals should be selective when deciding from whom and when to learn socially (Boyd and Richerson 1985; Laland 2004; Mesoudi 2008). In some species, individuals preferentially learned socially from successful models (Schwab et al. 2008; Duffy et al. 2009). For example, in the foraging-box experiment in vervet monkeys, bystanders paid more attention to female than male demonstrators, probably because they are the philopatric sex and may have more detailed knowledge about the distribution of food resources in their territory (van de Waal et al. 2010). The inventor in the open group B exhibiting a group preference was a 12-year-old female, whereas the inventor in the open group F with no group preference was a 2-year-old male. The group preference in the open group B might have been established because the inventor was older and a philopatric female, suggesting that learning might have been indirectly biased by favouring successful over less successful individuals (Boyd and Richerson 1985; Wilkinson 1992). Thus, these first indications for a group preference set the stage for further experimental studies of conformity in wild lemurs.

In conclusion, wild redfronted lemurs are able to learn new foraging techniques, and the use of social information facilitated the acquisition of new behaviours. Additionally, redfronted lemurs appear to prefer the technique that was used by the majority of the group. Because lemurs evolved group-living independently from other primates (Kappeler 1999) and represent the most basal living primates, they present an important model for establishing a baseline for social cognition to understand the evolution of culture in primates (Fichtel and Kappeler 2010). Since lemurs have been largely ignored in the field of social cognition and recent studies in this domain (Hosey et al. 1997; Fichtel and van Schaik 2006; Ruiz et al. 2009; Kendal et al. 2010b; Fichtel and Kappeler 2011; Stoinski et al. 2011) have revealed that they are more skilled than previously suggested (Jolly 1966; Deaner et al. 2007), this study also provides important new insights into our understanding of lemurs cognitive abilities in the social domain.
